# miR-25 mediates metastasis and epithelial–mesenchymal-transition in human esophageal squamous cell carcinoma via regulation of E-cadherin signaling

**DOI:** 10.1080/21655979.2019.1687391

**Published:** 2019-11-25

**Authors:** Bing Liu, Xuhua Li, Chuan Li, Ruixia Xu, Xuerong Sun

**Affiliations:** a Department of Thoracic surgery, The central hospital of Linyi, Linyi, Shandong, China; b Department of Thyroid surgery, The affiliated hospital of Qingdao University, Shandong, China; c Department of Thoracic surgery, The affiliated hospital of Qingdao University, Shandong, China

**Keywords:** Esophageal squamous cell carcinoma, lymph node metastasis, epithelial–mesenchymal transition, miR-25, E-cadherin

## Abstract

MiR-25 is a well-documented oncogenic miRNA implicated in esophageal squamous cell carcinoma (ESCC) development, progression and metastasis. However, whether and how miR-25 is involved in the development and metastasis of ESCC remain un-addressed. By using qRT-PCR analysis to compare levels of miR-25 in ESCC tissues with or without lymph node metastasis (LNM), it showed that ESCC tissues with LNM had increased levels of miR-25, which was correlated with tumor metastasis and poor prognosis. Gain- and loss-of-function assays indicated that targeting miR-25 could reverse EMT, and reduce in vitro cell migration and invasion, but not apoptosis and proliferation of ESCC. Furthermore, targeting miR-25 inhibited *in vivo* lung metastasis, and vice versa. And E-cadherin was a direct target of miR-25 through which affected EMT process and metastasis of ESCC. It is therefore indicated that miR-25 promotes metastasis of ESCC through E-cadherin and EMT events, thus may serves as a negative prognostic factor and possible target for treatment of ESCC patients.

## Introduction

Esophageal squamous cell carcinoma (ESCC) accounts for about 90% of the 456,000 incident esophageal cancers each year [1]. It is also the most common type of esophageal cancer in China, which accounts for more than 90% of cases [2]. The majority of esophageal cancer patients are diagnosed with advanced disease due to unclear early symptoms. The prognosis is very poor, with 5-year survival of only 10%, due to early invasion and metastasis via the well-developed network of submucosal lymphatic vessels [3]. Although previous studies have demonstrated that alterations of numerous oncogenes and tumor-suppressor genes are involved in ESCC, the underlying molecular and genetic mechanism of esophageal carcinogenesis remains largely unknown.

Numerous studies have indicated that microRNAs (miRNAs) are involved in multiple cellular processes as post-transcriptional regulators and particularly in cancer development and progression [4,5]. miRNAs are aberrantly expressed in various cancers and function as a novel class of oncogenes or tumor-suppressor genes depending on their targets. MiR-25 was reported to be associated with tumor carcinogenesis and metastasis, including ESCC [6–8]. But its exact mechanisms and roles *in vivo* are unclear.

Epithelial–mesenchymal transition (EMT) is considered to play an important role in the initial parts of the metastatic cascade-providing cancer cells with invasive, migratory, and cancer stem cell properties, and the induction of EMT leads to the downregulation of E-cadherin, expression of distinct mesenchymal markers such as vimentin, fibronectin, and N-cadherin, and morphological changes [9,10]. Several miRNAs, such as miR-200 family members, have been found to regulate EMT by targeting the E-cadherin repressors ZEB1 and ZEB2. MiR-124, a novel tumor-suppressor miRNA that is epigenetically silenced in EC, can reverse EMT and the invasive properties, by attenuating the expression of the IQGAP1 oncogene [11]. In HK-2 cells, enhanced expression of miR-106b attenuated EMT by retaining the epithelial morphology of HK-2 cells, reducing the levels of α-smooth muscle actin (α-SMA), and increasing the levels of E-cadherin [12]. In cervical cancer cells, miR‐25‐3p is an important regulator of cervical cancer EMT and chemoresistance [13].

In the present study, we investigated whether the relative expression of miR-25 between tumor and normal tissues is correlated with lymph node metastasis in ESCC patients and the mechanism by which miR-25 promotes ESCC metastasis and the same in turn. The results showed that high miR-25 expression is correlated with tumor metastasis and poor prognosis in ESCC patients. Targeting miR-25 could reverse EMT and reduce *in vitro* cell migration, invasion and *in vivo* lung metastasis, the mechanisms of which were by miR-25-dependent E-cadherin regulation. Thus, targeting miR-25 could be a novel approach to inhibit metastasis in patients with ESCC.

### Material and methods

#### Tissue specimens

Seventy-nine cancer tissues and adjacent normal tissues were obtained from ESCC patients who underwent surgery at the central hospital of Linyi, Shandong, China. The protocol was approved by the Ethics Committee of the hospital, and all experiments were conducted after acquiring informed consent. All samples were separated into two parts. One part was collected, snap-frozen immediately, and stored at −80°C for RNA isolation using TRIzol reagent (Invitrogen, Thermo Fisher Scientific). Another part was detected by immunohistochemistry probed with antibodies against E-cadherin (Signaling Technology Inc, Danvers, MA, USA).

#### Cell culture

The ESCC cell lines KYSE-410, Ec-109, CaEs-17 and KYSE-510 were obtained from DSMZ, the German Resource Center for Biological Materials and maintained in RPMI-1640 (**Life Technologies, Shanghai, China**) supplemented with 5% fetal calf serum at 37°C in a humidified atmosphere containing 5% CO_2_ and 95% air. All cell lines have been DNA fingerprinted using the PowerPlex 1.2 kit (P**romega, Beijing Biotech Co., Ltd., Beijing, China**) and are mycoplasma free using the e-Myco kit (**Boca Scientific, Florida, USA**).

#### Stable cell line selection

A pre-miR‐25 expression vector was constructed using a BLOCK‐iT™Pol II miR RNAi Expression Vector Kit (**Invitrogen, Shanghai, China**) according to the manufacturer’s protocol and transfected into the Ec-109 cells for selection of stable miR‐25‐expressing cells. After 48 h of transfection, cells were incubated with 10 mg/mL blasticidin for 2 weeks. To construct stably expressing anti-miR‐25 cells, an anti-miR‐25 expression vector was transfected into the KYSE-510 cells. After 48 h of transfection, cells were incubated with 2 mg/mL puromycin for 2 weeks. Then, cells were frozen in aliquots for later use.

#### Transfection–transduction

Transient transfection of Ec-109 or KYSE-510 cells using Lipofectamine 2000 was performed with pre-miR-25 or anti-miR-25 or negative control pre-miR (anti-miR) according to the manufacturer’s instructions. At 72 h after transfection, cells were counted and the cellular lysates were collected for analysis of the protein expression of the selected putative miR-25 targets. To study the effects of miR-25 on E-cadherin expression, the anti-miR-25 transfected KYSE-510 cells were seeded at 2 × 10^5^ cells per well in a 6-well plate and transfected with E-cadherin siRNA. After 48 h, cells were counted and lysed for RNA and protein extraction.

#### Quantitative RT-PCR

RNA was isolated from human tissue and cells using Trizol (**Life Technologies, Shanghai, China**) according to manufacturer’s protocol. miR qPCR was performed using Express SYBR GreenER Supermix with premixed ROX (**Invitrogen, Shanghai, China**). U6 was used as housekeeping gene for miR to normalize the Ct values (ΔCt), and relative expression was calculated using the 2^−ΔΔCt^ method. The 75^th^ percentiles of 2^ΔΔCt^ were used as the cutoff point for patients with high and low levels of miR-25. Primers for miR-25 were purchased from Qiagen **(Shanghai, China)** and relative expression was calculated using the 2^−ΔΔCt^ method.

#### Western blot analysis

Cells were harvested and washed with PBS twice, disrupted in IP buffer (Thermo) and centrifuged at 12,000 × g for 20 min. Protein (50 µg) from the supernatant fraction (quantified by the BCA Protein Assay Kit, Thermo) was subjected to SDS-PAGE, and transferred to a PVDF membrane (**Millipore, Guangzhou, China**). Membranes were blocked with 5% nonfat milk for 1 h at room temperature and then incubated with the anti-cadherin (**Abcam, Shanghai, China**), followed by the corresponding HRP-conjugated anti-mouse or anti-rabbit secondary antibody (**Abcam, Shanghai, China**). Protein bands were visualized by the Western lightening plus-ECL kit (**Pierce, Wuhan, China**).

#### Immunofluorescence

Cells grown on glass coverslips were fixed in 4% paraformaldehyde for 10 min at room temperature. Cells were washed twice with PBS. Blocking buffer (**DakoCytomation, Glostrup, Denmark**) was added for 30 min, and samples were then stained with primary E-cadherin antibodies (**Santa Cruz Biotechnology, Shanghai, China**) and double-stained with DAPI to reveal the nuclei. Images were taken using a fluorescence Nikon Ti-E inverted microscope (**MP110, Molson Pavillion**).

#### Cell migration and invasion assays

Cell migration and invasion assays were analyzed using the Transwell chambers assay (**Costar, Corning Inc., Corning, NY, USA**), with or without coated Matrigel (**BD Biosciences, San Jose, CA, USA**). Cells were plated at a density of 5 × 10^4^ per well in the upper chamber without serum. The lower chamber of the Transwell device was filled with 500 µl RPMI 1640 supplemented with 10% fetal bovine serum. After incubation for 24 h, noninvading cells were removed from the top well with a cotton swab, whereas the bottom cells were fixed with 3% paraformaldehyde, stained with 0.1% crystal violet, and photographed in three independent fields for each well. They were finally extracted with 33% acetic acid and detected quantitatively using a standard microplate reader (at 570 nm). Three independent experiments were conducted in triplicate.

#### Wound healing assays

A total of 100,000 Ec-109 or KYSE-510 cells were grown to 70% confluency in a 6-well plate (Falcon Becton Dickinson). The cells were transfected with pre-miR-25/pre-miR (Ec-109) and anti-miR-25/anti-miR (KYSE-510). After 5 h of transfection, 10% DMEM was added to cells. The cells were then serum-starved accordingly and three separate scratches were made per well after 24 h of transfection. The cells were left in serum-free or complete media for 48 h and the wounds were imaged at 200× magnification using Evos FL Digital Fluorescence microscope. The data is representative of two independent experiments done in triplicate.

#### Cell proliferation and apoptosis assay

To measure cell proliferation, cells were plated at a density of 1 × 10^3^ cells per well into 96-well plates at day 0 (24 h after pre-miR-25 or anti-miR-25 transfection). At days 1, 3 and 5, cell viability was measured using MTT as the manufacture’s instruction.

#### Immunohistochemical staining

Tissue sections were dewaxed with xylene and rehydrated through gradient ethanol into water. Antigen retrieval, lockage of endogenous peroxidase, and nonspecific binding were conducted before primary antibody incubation. Negative controls were obtained by omitting the primary antibody. Expression E-cadherin were evaluated as described previously. The percentage of positive tumor cells was determined semi-quantitatively by assessing the entire tumor section. Each sample was assigned to one of the following categories: 0 (0–4%), 1 (5–24%), 2 (25–49%), 3 (50–74%), or 4 (75–100%). The intensity of immunostaining was determined as 0 (negative), 1+ (weak), 2+ (moderate), or 3+ (strong). A final immunoreactive score between 0 and 12 was calculated by multiplying the percentage of positive cells with the staining intensity score. The two scores were then multiplied to produce a weighted score for each sample, the expression was considered positive when the score was >2. All slides were blindly evaluated for immunostaining without any knowledge of the clinical outcome of other clinical or pathological data.

#### Metastasis assays in nude mice

For lung metastasis model, 2 × 10^6^ cells (stable Ec-109/pre-miR-25, KYSE-510/anti-miR-25 and their controls) of each group were washed in PBS and injected intravenously into 4- to 6-week-old age-matched female. Animals were sacrificed 4 weeks after injection and the lung surface tumor foci were counted. Lung tissues were fixed in formalin and sectioned for H&E staining.

#### Statistical analysis

All data were expressed as mean ± standard deviation, and SPSS 22.0 software was used for statistical analyses. All *in vitro* experiments were repeated at least three times unless stated otherwise. Differences among the groups and treatments were determined by Student’s *t*-test or one-way ANOVA unless stated otherwise. Survival was estimated using the Kaplan–Meier method. Significance between survival functions was identified by the log-rank test. *P*< 0.05 was considered statistically significant.

## Results

### Expression of miR-25 is closely associated with lymph node metastasis and poor survival

**Figure 1.**
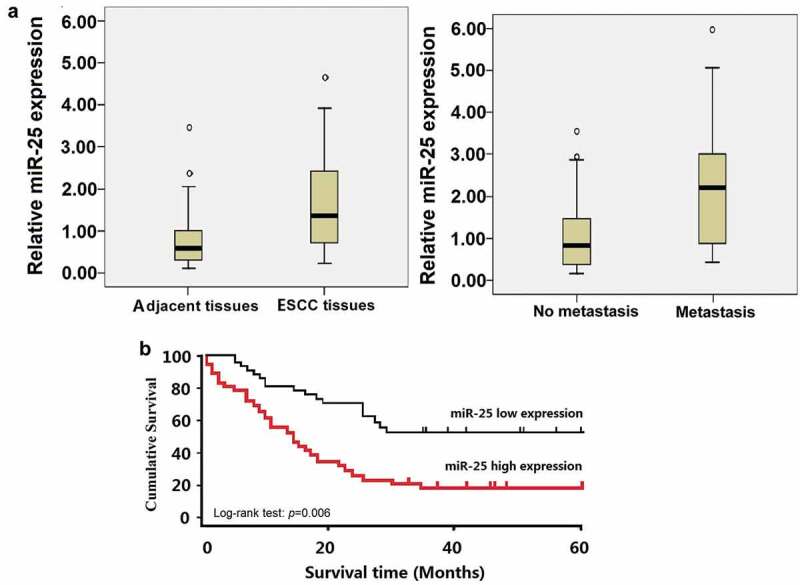
Expression of miR-25 in ESCC tissues and its relation with survival.

Seventy-nine ESCC tissues and neighboring non-cancerous tissues were assessed for miR-25 expression level by qRT-PCR. Quantitative data indicated that the levels of miR-25 in ESCC tissues were notably elevated compared to the matched non-cancerous tissues (t = 4.673, P = 0.0026, Figure 1(a)a). Furthermore, the levels of miR-25 were significantly higher in metastatic tumor tissues than that in the primary tumor tissues without detectable metastasis (t = 4.156, P = 0.0163, Figure 1(a,b) (Table 1). The 75th percentiles of 2^ΔΔCt^ was used as the cutoff point for patients with high and low levels of miR-25. Kaplan–Meier survival estimate showed that the patients with high miR-25 expression (n = 34) had a shorter survival compared with the patients with low miR-25 expression (n = 45). The difference in the prognosis of these two groups was statistically significant (p = 0.006, log-rank test, Figure 1(b)).

**Table 1.** Correlation between E-cadherin, lymph node metastasis and miR-25 expression.

			miR-25 expression		
Features		Number	Low	High	p-value
E-cadherin	Negative	60	33	27	0.0016
	Positive	19	12	7	
Lymph node metastasis	No	35	21	14	0.028
	Yes	44	24	20	

The industry composition follows the CSRC’s industry coding system in 2001. Manufacturing (labeled C) is divided into ten categories because more than 60% of the sample firms are manufacturers. We omit the banking and insurance industry (labeled I) in our sample.

### miR-25 modulates migration and invasion but not proliferation in ESCC cells

**Figure 2.**
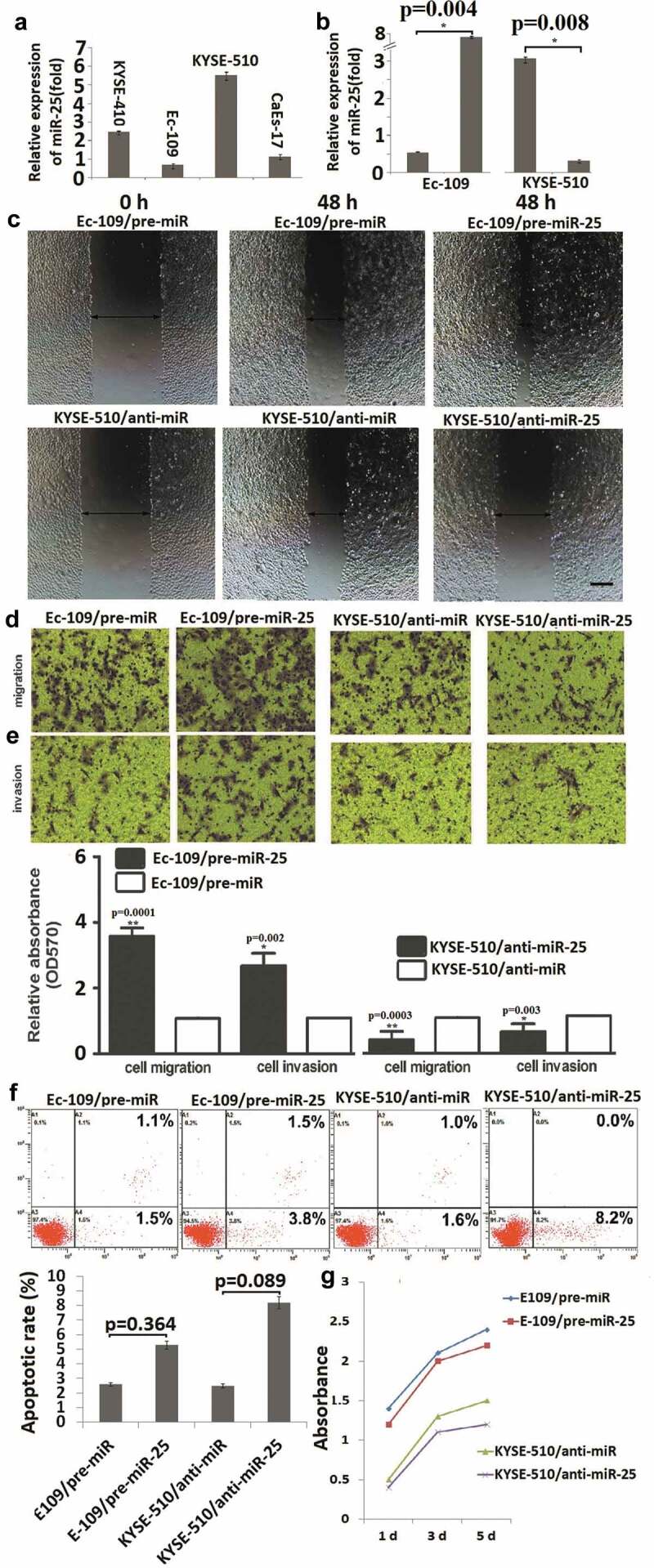
MiR-25 regulated the migration, invasion but not apoptosis in ESCC cells.

In the pre-experiment, miR-25 expression was detected by qRT-PCR in KYSE-410, Ec-109, CaEs-17 and KYSE-510 cells, showing that Ec-109 cells have the lowest miR-25 expression, and KYSE-510 has the highest miR-25 expression (Figure 2(a)). So the Ec-109 and KYSE-510 cells were used for further study. To further investigate the biological mechanism of miR-25 on the invasiveness of ESCC, *in vitro* gain- and loss-of-function analysis was carried out through transient transfection in ESCC cells. miR-25 overexpression experiments were first performed by miR-25 precursor (pre-miR-25) transfection in the Ec-109 cell line. Overexpression of miR-25 was confirmed by qRT-PCR, as shown in Figure 2(b) (**P = 0.004**). In the wound healing assay, high miR-25 expression significantly promotes the ability of cells to migrate (Figure 2(c)). In the transwell invasion and migration assay, cells transfected with pre-miR-25 displayed an increase in invasion and migration ability when compared with the control groups (Figure 2(d–e)). Collectively, these results indicated that ectopic miR-25 significantly promoted cell migration and invasion in vitro.

To be logical and direct demonstration of the consequences of the low expression levels of miR-25 in ESCC, miR-25 inhibition experiments were performed by miR-25 inhibitor (anti-miR-25) transfection in the KYSE-510 cell line. miR-25 was transiently inhibited in KYSE-510 cells with anti-miR-25 (P = 0.008, Figure 2(b)), and the effect of inhibition of miR-25 levels on cellular invasion and migration potential of KYSE-510 cells was analyzed. The results showed that the suppression of miR-25 decreased invasion and migration in KYSE-510 cells (Figure 2(c–e)). However, overexpression of miR-25 in Ec-109 cells (**P = 0.364**) and knock-down of miR-25 in KYSE-510 cells (**P = 0.089**) had no significant effect on cell apoptosis (Figure 2(g)) and proliferation (Figure 2(h)). These results demonstrated that miR-25 promotes ESCC cell migration and invasion but has no effect on cell apoptosis and proliferation.

### Expression of miR-25 is closely associated with E-cadherin

**Figure 3.**
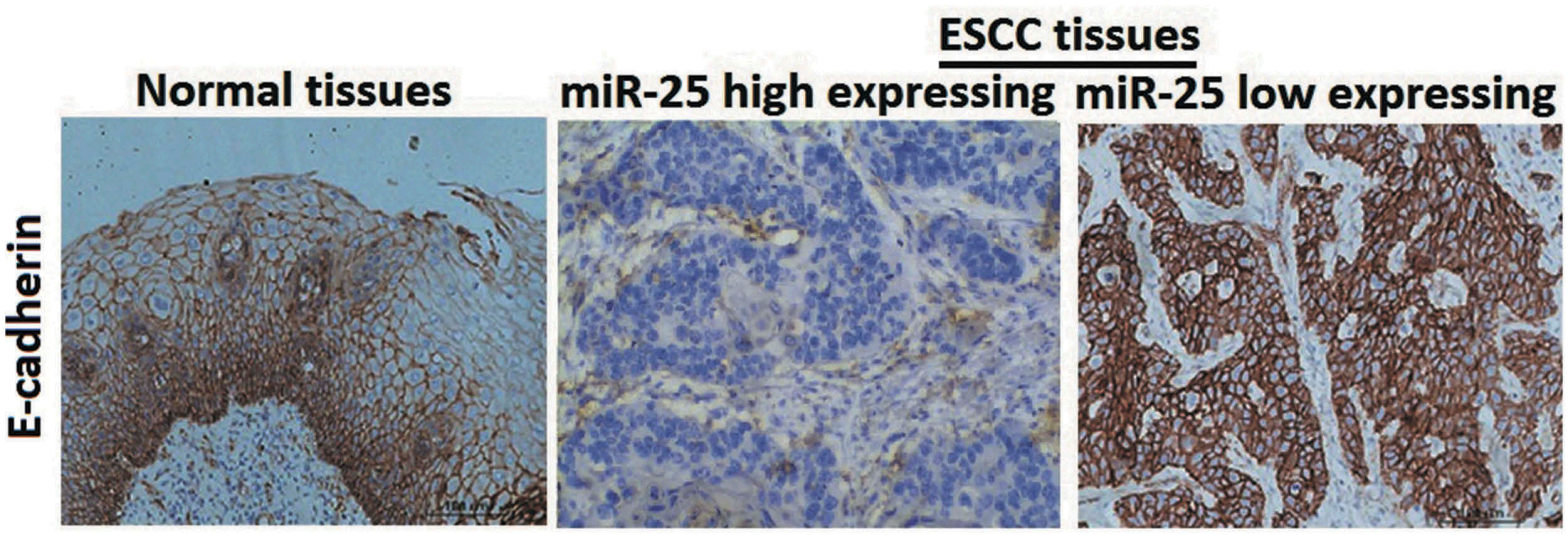
Immunohistochemical staining of E-cadherin.

Immunohistochemical staining showed that E-cadherin was strongly expressed in the membrane in non-cancerous tissues (100%, 79/79), while the positive expression rate was significantly lower in ESCC (19/79). Representative immunostaining showed that ESCC tissues with high expression of miR-25 had\weak staining of E-cadherin, and ESCC tissues with low expression of miR-25 had strong staining for E-cadherin (Figure 3). Statistical analysis showed that the expression of miR-25 was closely related with E-cadherin (P = 0.0016) (Table 1). The results taken together revealed that miR-25 expression was inversely correlated with E-cadherin expression.

### miR-25 regulates E-cadherin expression in ESCC cells

**Figure 4.**
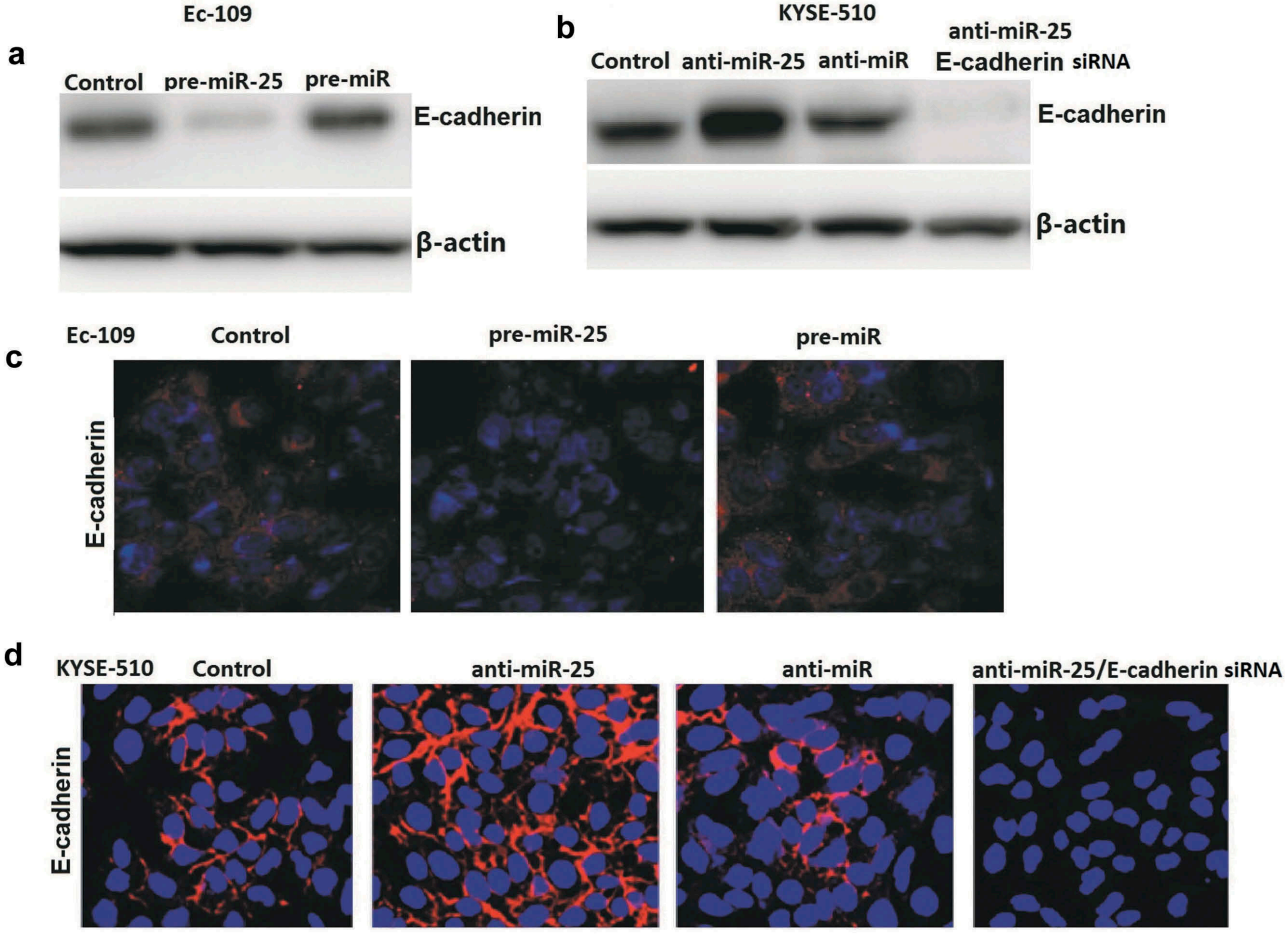
Effect of miR-25 on E-cadherin genes in Ec-109 and KYSE-510 cells.

To determine whether miR-25 regulates E-cadherin -induced EMT in ESCC cells, the effect of miR-25 on E-cadherin expression regulation was analyzed. Ec-109 cells were transfected with 100 nM pre-miR-25 or pre-miR control for 48 h, and KYSE-510 cells were transfected with 100 nM anti-miR-25 and anti-miR control for 48 h. The results showed that pre-miR-25 treatment inhibits E-cadherin expression in Ec-109 cells (Figure 4(a)). And anti-miR-25 treatment promotes E-cadherin expression in KYSE-510 cells (Figure 4(b)). Consistent with the western blot assay, immunofluorescence staining by confocal laser scanning microscope for E-cadherin showed the same results (Figure 4(c,d)).

### E-cadherin is crucial for miR-25-mediated EMT

**Figure 5.**
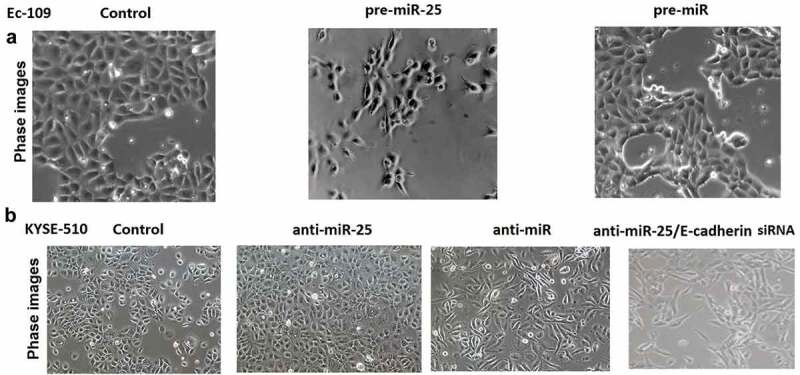
Down-regulation of miR-25 or up-regulation of miR-25 in cells having undergone EMT.

Untreated Ec-109 and KYSE-510 cells had an almost round shape under the phase-contrast microscopy (Figure 5). In Ec-109 cells, miR-25 transfection replaced cobblestone-like appearance with spindle-shaped cells like the mesenchymal cells. In KYSE-510 cells, anti-miR-25 transfection makes cobblestone-like appearance with more dense cell-to-cell adhesions and assembled closely, whereas anti-miR-25 + E-cadherin siRNA treated KYSE-510 cells developed a spindle-shaped morphology, the cell-to-cell adhesions became weak and the cells were scattered compared to the anti-miR-25 alone treated KYSE-510 cells (Figure 5).

### Upregulation of miR-25 induced lung metastasis, and targeting miR-25 inhibited lung metastasis in mice

**Figure 6.**
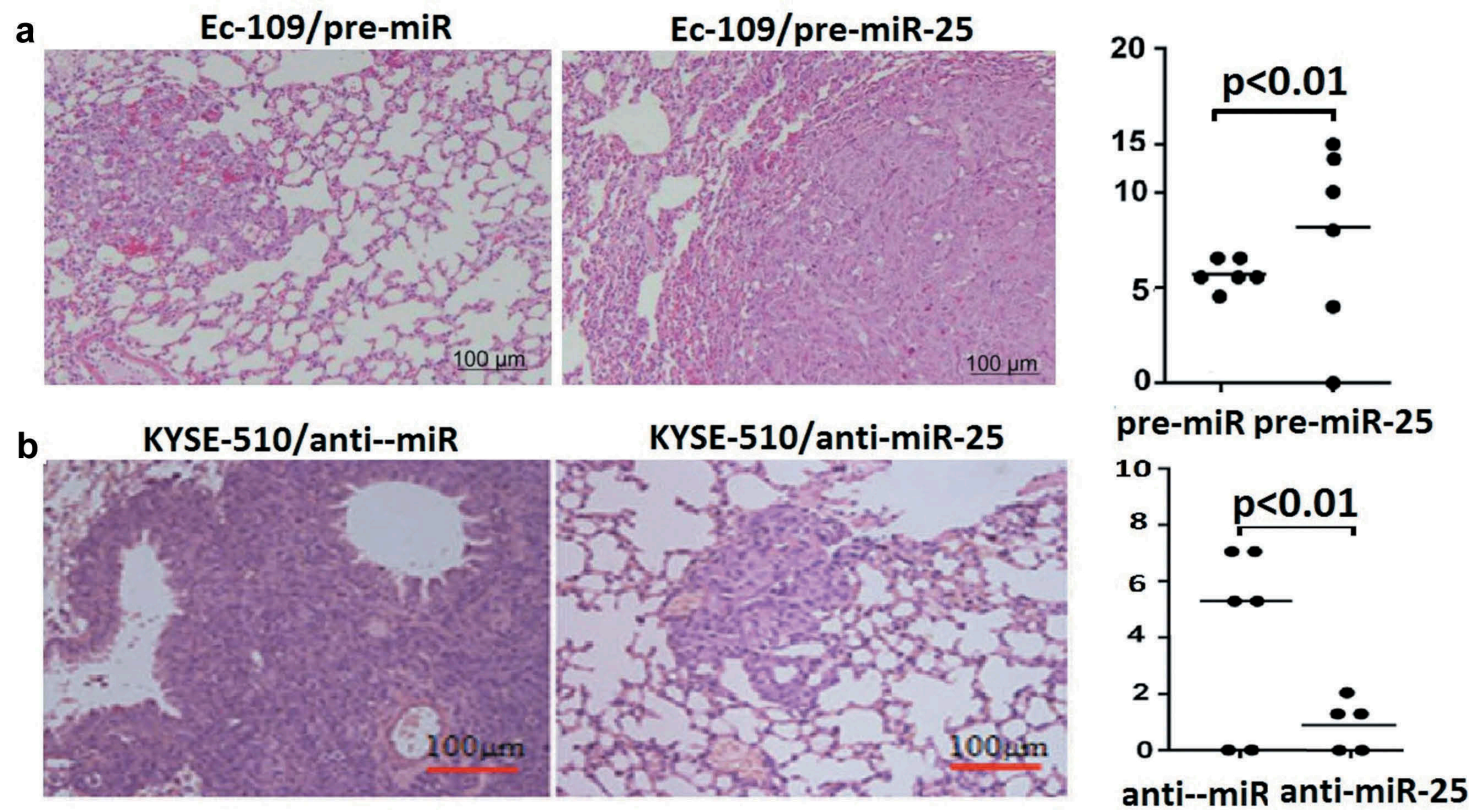
Effect of miR-25 lung metastasis in mice.

In Ec-109 cells and KYSE-510 cells, the vector treated cells (Ec-109/pre-miR and KYSE-510/anti-miR) formed multiple and large-sized metastatic nodules in the lung (Figure 6(a-b)). In Ec-109 cells, more lung metastatic nodules were observed in the lungs of the pre-miR-25 expression groups compared with the control group (Figure 6(a)). However, in KYSE-510 cells, fewer lung metastatic nodules were observed in the lungs of the anti-miR-25 expression group compared with the control group (Figure 6(b)).

## Discussion

In the present study, miR-25 is upregulated in the ESCC tissues than the matched non-cancerous tissues. In addition, miR-25 is more strongly upregulated in the metastatic ESCC tissues than that in primary tumor tissues without detectable metastasis. High miR-25 expression was significantly associated with lymph node metastasis and poor overall survival and was confirmed as an independent prognostic factor in multivariate analysis. miR-25 is independent of traditional clinicopathological factors, including patient sex, age, clinical stage, tumor differentiation, and tumor grade. Therefore, miR-25 showed correlated mRNA over-expression in ESCC development and progression, and could be a diagnostic biomarker and gene target. Our results are agreed with the previous study in ESCC tissues [8], but contrary to Wang’s report that overexpression of miR-25 showed a positive correlation with overall survival in ESCC patients [14]. Besides, it was also revealed that miR-25 is a critical positive regulator of EMT program mainly through affecting the E-cadherin, and it promotes ESCC cells migration and invasion *in vitro* and *in vivo*, and targeting miR-25 inhibits ESCC cells migration and invasion in vitro and in vivo.

Metastasis is the primary cause of mortality in most cancer patients [15]. Thus, to understand the molecular mechanisms of metastasis is one of the most important issues in cancer research. Epithelial–mesenchymal transition (EMT), which enables epithelial cells to acquire invasive mesenchymal phenotype, is attracting increasing attention as an important mechanism for the initial step of metastasis [16,17]. It has been proven that loss of expression and/or abnormal function of E-cadherin leads to loss of cell polarity and derangement of normal tissue architecture. In most cancers with epithelial origins, E-cadherin -mediated cell-cell adhesion is lost concomitantly with the acquisition of an invasive phenotype, high tumor grade, and low patient survival [18–20]. Loss or reduction of E-cadherin expression can be caused by a variety of mechanisms, such as somatic mutations, chromosomal deletions, proteolytic cleavage, and silencing of the E-cadherin promoter [21]. Our study revealed a novel mechanism by which E-cadherin is regulated. Our study showed that E-cadherin is directly targeted by miR-25 in ESCC cell lines, and an inverse correlation between miR-25 and E-cadherin in ESCC tumor tissues and cell lines was uncovered. Furthermore, restoration of E-cadherin expression inhibited cell invasion and migration induced by miR-25 overexpression, and targeting E-cadherin reversed cell invasion and migration induced by targeting miR-25, indicating that E-cadherin functions as a mediator of miR-25 in cell migration and ESCC progression.

EMT is a key factor to embryonic development and tumor metastasis. Our data demonstrated that miR-25 overexpression inhibited E-cadherin expression and induced EMT. However, targeting miR-25 induced E-cadherin expression and suppressed EMT, and targeting of E-cadherin in cancer cells could be sufficient to reverse EMT in anti-miR-25 treated cells, which was accompanied by a more invasive property. Our findings showed that miR-25 is required for EMT initiation and maintenance, which was related to E-cadherin regulation, and a common regulatory mechanism of tumor cell invasion.

### Conclusions

Our study not only revealed the important role of miR-25/E-cadherin signaling pathway in ESCC pathogenesis, but also implied a potential role for miR-25 in the clinical diagnosis and treatment of ESCC. Our study also suggests that miR-25 is required for EMT initiation and maintenance. miR-25 plays an important role in EMT through direct targeting of E-cadherin. Therefore, miR-25 could be used as effect target for ESCC treatment.

## Highlight

- High miR-25 expression is correlated with metastasis in patients with esophageal squamous cell carcinoma.
- High miR-25 expression is correlated with poor prognosis in patients with esophageal squamous cell carcinoma.
- Targeting miR-25 could reverse epithelial–mesenchymal transition in esophageal squamous cell carcinoma cells in vitro.
- Targeting miR-25 reduces *in vitro* cell migration, invasion and *in vivo* lung metastasis.
- miR-25 inhibits invasion and metastasis of esophageal squamous cell carcinoma cells through E-cadherin upregulation.
